# Psychosexual Consequences of Female Genital Mutilation and the Impact of Reconstructive Surgery: A Narrative Review

**DOI:** 10.1089/heq.2018.0036

**Published:** 2019-02-20

**Authors:** Laura Buggio, Federica Facchin, Laura Chiappa, Giussy Barbara, Massimiliano Brambilla, Paolo Vercellini

**Affiliations:** ^1^ Gynaecology Unit, Fondazione IRCCS Ospedale Maggiore Policlinico, Milan, Italy.; ^2^ Faculty of Psychology, Catholic University of Milan, Milan, Italy.; ^3^ Health Director, Fondazione IRCCS Ca’ Granda Ospedale Maggiore Policlinico, Milan, Italy.; ^4^ Department of Obstetrics and Gynecology and Service for Sexual and Domestic Violence (SVSeD), Fondazione IRCCS Ca’ Granda Ospedale Maggiore Policlinico, Milan, Italy.; ^5^ Plastic Surgery Unit, General Surgery Department, Fondazione IRCCS Ca’ Granda Ospedale Maggiore Policlinico, Milan, Italy.; ^6^ Department of Clinical Sciences and Community Health, Università degli Studi Milano, Italy.

**Keywords:** clitoral reconstruction, female genital cutting, female genital mutilation, FGM/C, psychological complications, sexual function

## Abstract

**Purpose:** We aim to provide a comprehensive overview of the health consequences of female genital mutilation/cutting (FGM/C), with a particular focus on the psychosexual implications of this practice and the overall impact of reconstructive plastic surgery.

**Methods:** A MEDLINE search through PubMed was performed to identify the best quality evidence published studies in English language on long-term health consequences of FGM/C.

**Results:** Women with FGM/C are more likely to develop psychological disorders, such as post-traumatic stress disorder, anxiety, somatization, phobia, and low self-esteem, than those without FGM/C. Most studies showed impaired sexual function in women with FGM/C. In particular, women with FGM/C may be physiologically less capable of becoming sexually stimulated than uncut women. Reconstructive surgery could be beneficial, in terms of both enhanced sexual function and body image. However, prospective studies on the impact of reconstructive surgery are limited, and safety issues should be addressed.

**Conclusion:** Although it is clear that FGM/C can cause devastating immediate and long-term health consequences for girls and women, high-quality data on these issues are limited. Psychosexual complications need to be further analyzed to provide evidence-based guidelines and to improve the health care of women and girls with FGM/C. The best treatment approach involves a multidisciplinary team to deal with the multifaceted FGM/C repercussions.

## Introduction

Female genital mutilation/cutting (FGM/C) includes all procedures that involve intentional removal (either partial or total) of external female genitalia for nonmedical reasons and without health benefits.^1^ This practice violates a series of human right principles, and may cause several immediate and long-term consequences.^1^

Despite the implementation of laws prohibiting this practice, FGM/C is still performed in ∼30 African countries and in a few Asian and Middle East countries.^2,3^ Moreover, the prevalence of girls and women with FGM/C is also rising in Western countries due to migration flows.^4^ As reported by Farina et al.,^5^ ∼57,000 foreign girls and women with FGM/C aged between 15 and 49 were living in Italy in 2010. Worldwide, it is estimated that ∼200 million girls and women have undergone FGM/C,^6^ and >3 million girls are at risk of cutting every year.^7^

FGM/C has been practiced for centuries and has established its roots in ancient sociocultural traditions, which vary from one region and ethnic group to another. Reasons for performing FGM/C include social acceptance, the safeguard of virginity before marriage, and the promotion of marriageability.^1^ In addition, in some communities, cutting is seen as a rite of passage to adulthood, and is part of the history and cultural tradition of the specific ethnic group.^1^ Contrary to common belief, neither the Koran nor the Bible condones this procedure.^1,8^ However, religious interpretations have been used to justify FGM/C in some communities.^8^

FGM/C is a deeply entrenched social norm, and its eradication appears extremely difficult. Nevertheless, in 2009 the majority of the African states where this practice is performed have adopted laws against FGM/C^9^ and 18 countries developed a national legislature against genital mutilation.^10^ In 2012, 194 United Nations member states approved a resolution aimed at raising awareness and allocating adequate resources to protect and support women victims of FGM/C.^11^ Legal frameworks are essential elements of a comprehensive response aimed at abolishing this practice^10^; in this regard, positive change is now happening in terms of slow decrease of global FGM/C rates.^12^ However, there is also need for measures addressing the underlying sociocultural traditions that are the core of this practice.^10^

Another fundamental aspect is the need for adequate health management of women with FGM/C, which frequently present various long-term health consequences, including gynecological, obstetrical, urological, psychological, and sexual complications.^1^ In this narrative review, we aimed to provide a comprehensive overview of the health consequences of FGM/C, with a particular focus on the psychosexual implications of this practice and the overall impact of reconstructive plastic surgery.

## Materials and Methods

For this review, the best quality evidence was selected with preference given to the most recent and definitive original articles and reviews. Information was identified by searches of PubMed/MEDLINE and references from relevant articles. We selected appropriate research terms by reviewing keywords, titles, and abstracts of a sample of studies. “Female genital mutilation,” “complications,” “obstetric outcome,” “reconstructive surgery,” “plastic surgery,” “health problems,” “mental problems,” “sexual function,” “sexual problems,” “psychological impact,” and “clitoral reconstruction” were used and combined as research terms. The search was limited to full-text articles in the English language. For most issues, papers published between November 1983 and April 2018 were considered. No attempt was made to find unpublished studies. Since only published data were considered, the current research project was exempt from Institutional Review Board approval, and informed consent was not obtained because we did not recruit any human subject.

## FGM/C Classification

**FIG. 1.**
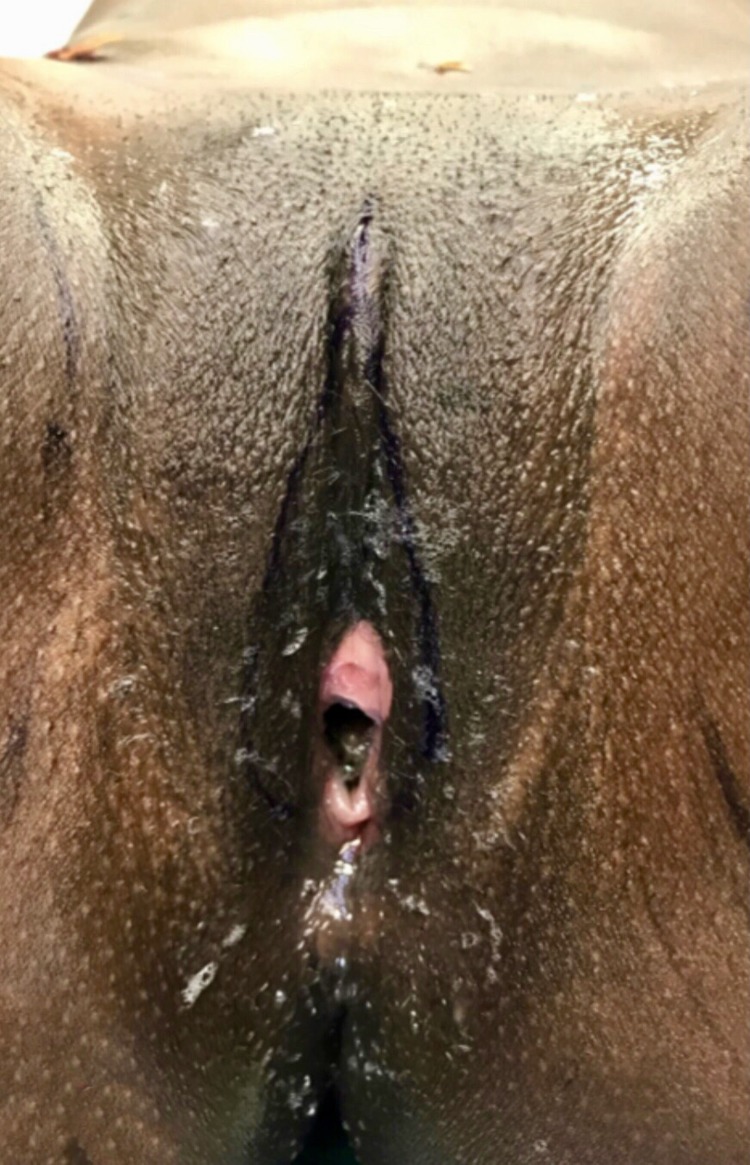
Twenty-three year old nulliparous woman with a female genital mutilation type II. © Massimiliano Brambilla 2018.

**FIG. 2.**
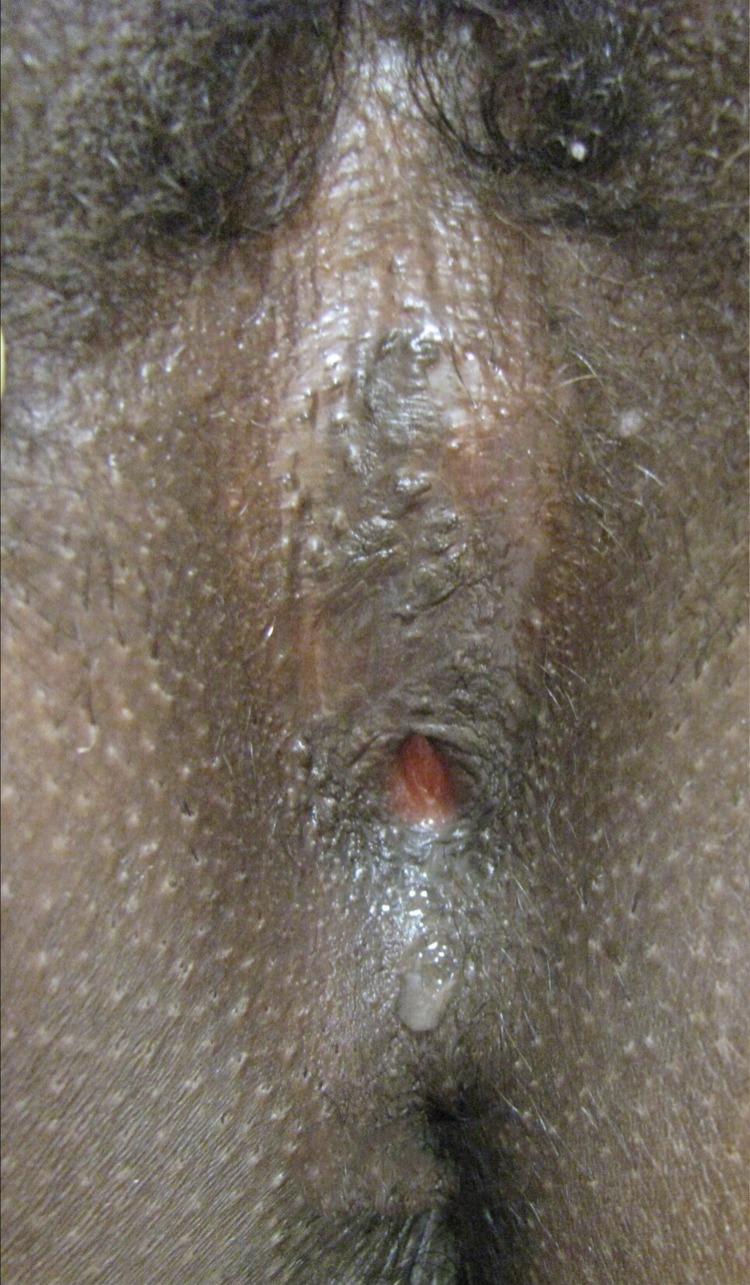
Twenty-one year old nulliparous woman with a female genital mutilation type IIIb. © Massimiliano Brambilla 2018.

The World Health Organization (WHO) has classified FGM/C into four categories (Table 1).^1^ Even if the WHO classification is very detailed, sometimes women's genital appearance may not fit precisely into one specific FGM/C type. To overcome this classification bias, UNICEF has proposed another simpler classification system (Table 1).^13^ The most common forms of FGM/C are types I and II, which account for the 80% of the procedures (Fig. 1), whereas infibulation (type III) is performed in 15% of cases (Fig. 2), although with great variability among countries.^1^

**Table 1.** The 2016 WHO and UNICEF Classification of Female Genital Mutilation^a^

WHO classification:^1^
*Type I*: Partial or total removal of the clitoris (clitoridectomy) and/or the prepuce
*Type Ia*: removal of the prepuce/clitoral hood (circumcision)
*Type Ib*: removal of the clitoris with the prepuce (clitoridectomy)
*Type II*: Partial or total removal of the clitoris and the labia minora, with or without excision of the labia majora (excision)
*Type IIa*: removal of the labia minora only
*Type IIb*: partial or total removal of the clitoris and the labia minora
*Type IIIc*: partial or total removal of the clitoris, the labia minora, and the labia majora
*Type III*: Narrowing of the vaginal orifice with the creation of a covering seal by cutting and appositioning the labia minora and/or the labia majora, with or without excision of the clitoris (infibulation)
*Type IIIa*: removal and appositioning the labia minora (with or without excision of the clitoris)
*Type IIIb*: removal and appositioning the labia majora (with or without excision of the clitoris)
*Type IV*: Unclassified; includes all other harmful procedures to the female genitalia for nonmedical purposes, for example: pricking, pulling, piercing, incising, scraping, and cauterization
UNICEF classification:^13^
*Type 1*: cut, no flesh removed/nicked
*Type 2*: cut, some flesh removed
*Type 3*: sewn closed
*Type 4*: type not determined/not sure/does not know

^a^ References^1,13^.

UNICEF, United Nations International Children's Emergency Fund; WHO, World Health Organization.

In the majority of cases, FGM/C is performed by a traditional practitioner, generally a woman, without any form of anesthesia and using nonsterile devices, such as scissors, broken glasses, or razor blades.^12^ This procedure is evidently associated with immediate and chronic health repercussions that are influenced by different cofactors, such as the physical condition of the girl undergoing the mutilation, the skill of the cutter, the cleanliness of the procedure, and especially the type of FGM/C executed.^1^ In particular, women who had endured a type III procedure present a double risk of complication compared with those who had undergone a type II mutilation.^14^

## Physical Consequences

Complications secondary to FGM/C are classified into short- and long-term consequences (Table 2), which also entail FGM/C psychological and sexological impact. In addition, women who have undergone FGM/C show a greater number of obstetric complications, particularly among those who deliver in poor-resource settings.^15–17^ Obstetric outcomes greatly vary depending on the study context. In a 2006 African multicenter study on 28,393 women with FGM/C, participants with genital mutilation were at increased risk of prolonged labor, postpartum hemorrhage, caesarean section (CS), and perineal trauma relative to women without FGM/C.^17^ The risks seemed to be greater in case of more extensive FGM/C. Moreover, infants born from a mother with FGM/C were at increased risk of stillbirth and early neonatal death, with FGM/C estimated to lead to an extra 1–2 perinatal deaths per 100 deliveries. The authors underlined the fact that this prospective study was performed in hospitals; because the majority of women who have undergone FGM/C live in countries with limited infrastructures for health care, which most of them are not able to afford, the study population may have over-represented a small selected group that could afford hospital care. Thus, the proportion of obstetrical complications in women with FGM/C might have been underestimated. On the contrary, a recent Australian descriptive study^18^ on 196 births from women with FGM/C showed comparable maternal and neonatal outcomes, except for statistically significant higher rates of first- and second-degree perineal tears and CS; however, none of the CS had FGM/C as an indication. Essen et al.^19^ did not confirm the association between prolonged labor and female circumcision. In 2009, a Swiss retrospective case–control study^20^ reported no differences in 122 FGM/C patients and 110 controls regarding the fetal outcome, maternal blood loss, or labor duration; nevertheless, women with FGM/C had significantly more often an emergency CS and third-degree vaginal tears. A 2013 systematic review^21^ of data derived from 28 comparative studies involving ∼3 million participants indicated that prolonged labor, obstetric lacerations, instrumental delivery, obstetric hemorrhage, and difficult delivery are related to FGM/C. The authors highlighted that the overall quality of the included studies was low, and thus encouraged additional research to further examine these associations.

**Table 2.** Health Risks Associated with Female Genital Mutilation

Short-term complications
Extreme pain
Hemorrhage
Shock (hemorrhagic, neurogenic, septic)
Infection (wound infection, septicemia, gangrene, tetanus, genital and reproductive tract infections, urinary tract infections, possible association with increased risk of HIV and HCV due to the use of the same surgical instrument without sterilization)
Necrotizing fasciitis
Acute urine retention, urethral injury
Death (secondary to severe bleeding or septicemia)
Long-term complications
*Urogynecological*: infections (chronic genital abscesses, recurrent vaginal infections, recurrent urinary tract infections), genital scarring, inclusion cysts, menstrual disorders (dysmenorrhea, irregular periods, and difficult passage of menstrual blood with the risk of hematocolpo), chronic vulvar and pelvic pain, painful urination, infertility (primary, ascending pelvic infections)
*Obstetrical*: increased risk of prolonged labor, postpartum hemorrhage (blood loss ≥500 mL), episiotomy, perineal trauma, caesarean section, instrumental delivery, prolonged hospitalization, stillbirth, and early neonatal death^a^
*Psychological*: post-traumatic stress disorder, anxiety, depression, memory problems
*Sexual complications*: dyspareunia (particularly with type III FGM), decreased sexual satisfaction and lubrication during intercourse, reduced sexual desire and arousal, increased risk of anorgasmia

^a^ Obstetrical risks referred to studies performed in low-income countries; studies performed in Western setting suggest that a high standard of obstetric care can minimize these complications.^15^

FGM, female genital mutilation.

The most accredited potential mechanism related to the augmented risk of obstetrical complications in women with FGM/C is the presence of inelastic pelvic and vaginal tissues, subsequent to an excessive and abnormal wound-healing process.^17^ In addition, FGM/C is associated with an increased rate of urinary and genital infections, which could negatively affect the obstetric outcome.^17^

## Psychological Consequences

As underlined by Behrendt and Moritz,^22^ FGM/C represents a violation of women's physical intactness. Moreover, it may cause psychological trauma to the point of developing a post-traumatic stress disorder (PTSD) according to the DSM-IV criteria,^23^ as well as to those of the more recent DSM-5.^24^ One should also consider that the Istanbul Convention adopted by the Council of Europe Committee of Ministers in 2011 recognized FGM/C as a form of gender-based violence.^25^ Despite the evident clinical and social relevance of the phenomenon, there is currently scanty research exploring the psychological impact of FGM/C.

There is evidence that women with FGM/C are more likely to experience anxiety, somatization, phobia, and low self-esteem than those without genital mutilation.^26^ In a pilot study on 47 Senegalese women, Behrendt and Moritz^22^ showed a significantly higher prevalence of PTSD (30.4%, *n*=7/23) and other psychiatric syndromes (47.9%, *n*=11/23) in 23 cut women (vs. 24 uncut participants). In addition, PTSD was associated with memory problems.^22^ The high percentage of PTSD (>30%) in women with FGM/C was comparable with that observed in cases of early childhood abuse (usually between 30% and 50%).^22^ These results are in line with those found by Chibber et al.^27^ in a cross-sectional study on 4800 pregnant women; more than half of women with FGM/C showed affective disorders, with PSTD rate up to 30%. Moreover, 85% of circumcised women reported the presence of emotional effects, such as flashbacks to the cutting event. A recent Dutch cross-sectional research^28^ on 66 genitally mutilated African women has confirmed a high rate of psychopathology in this category of women; in fact, a fifth of the respondents (20%, *n*=13) met the criteria for PTSD, a third met these for depression (33%, *n*=22), and nearly a third met those related to anxiety disorders (30%, *n*=20). Infibulation, vivid memory of the cutting event, lack of income, and avoidant coping style (especially when characterized by substance misuse) were significantly associated with psychopathology. A recent cross-sectional Egyptian study^29^ evaluated the psychological impact of FGM/C among 204 adolescent girls aged between 14 and 19 years, demonstrating a significantly higher level of somatization, depression, anxiety, phobic anxiety, and hostility compared with girls without FGM/C. On the contrary, an Israel case–control study^30^ failed to demonstrate an association between genital mutilation and psychological disorders. However, as stated by Berg et al.,^26^ the studies analyzing the psychological consequences of FGM/C are characterized by low-quality designs, small sample sizes, and inconsistent results, thus precluding the drawing of firm conclusions.

The WHO health consequences guidelines based on the existing evidence suggest that cognitive behavioral therapy (CBT), focused on the complex interaction between thoughts, affects, and behaviors, as well as on emotional regulation and stress reduction, may work with girls and women who experienced FGM/C with current symptoms of anxiety, depression, and/or PTSD.^1^ Although there are no studies directly investigating the effectiveness of CBT in women with FGM/C, this type of intervention was successfully used to treat PTSD following a variety of events, including torture, wars, and sexual violence.^31,32^ Psychological support is also indicated for women who decide to undergo surgery to correct health complications of FGM/C to avoid retraumatization. In fact, the surgical intervention itself and the pain experienced may remind women of the mutilation, to the point of triggering a relapse of PTSD symptoms.^33^ From this perspective, psychological support would be important for postoperative pain management, recovery, and psychological well-being.^1^

## Consequences on Sexual Function

Human sexuality results from the interaction of multiple anatomical, neurological, emotional, physiological, and biochemical mechanisms, and is influenced by sociocultural factors and relationship dynamics.^34,35^ In women with FGM/C, parts of the erogenous genital zones and sexually responsive vascular tissue are excised.^26^ The removal of women's genital parts may lead to damaged nerve endings, as well as to the development of inelastic scar tissue and adhesions surrounding the excised areas, and for this reason FGM/C may cause impaired sexual functioning.^36^

Berg and Denison^37^ performed a meta-analysis on the sexual consequences of FGM/C, showing that circumcised women were more likely to report dyspareunia (RR 1.52, 95% CI: 1.15–2.0), poor sexual satisfaction (standardized mean difference=−0.34, 95% CI=−0.56 to −0.13), and absence of sexual desire (RR 2.15, 95% CI: 1.37–3.36). In the past, one of the major drawbacks of the published articles was methodological; in fact, few studies have adopted validated questionnaires to analyze sexual function. To overcome this problem, in the last decade several studies have used a validated instrument, such as the Female Sexual Functioning Index (FSFI),^38^ to measure women's sexual functioning (Table 3). Except for one study,^39^ women with FGM/C showed lower scale scores, indicative of sexual dysfunction. These results are in line with those of Andersson et al.,^53^ who used the Sexual Quality of Life-Female (SQOL-F) questionnaire to investigate sexual function in women with FGM/C, as well as with those of Thabet and Thabet,^36^ who adopted a nonvalidated questionnaire developed by the authors themselves. On the contrary, Catania et al.^39^ performed a case–control study on 114 women showing comparable results between the two study groups regarding lubrication and pain subdomains, and infibulated women obtained significantly higher scores in desire, arousal, orgasm, and satisfaction subdomains. However, the fact that the control group was composed of 54 Western women, with only three from Somalia, may have affected the findings by making the two groups less comparable in terms of cultural background.

**Table 3.** Summary of Studies on Sexual Function That Have Adopted the Female Sexual Function Index as Evaluating Tool

Source	Study design	Country	Number of patients enrolled	FSFI full-scale score	Outcomes
Catania et al.^39^	Case–control study	Italy	114 (*n*=57 women with FGM/C; *n*=57 women without FGM/C)	N.A.	No significant differences between the two groups in lubrication and pain domains. Infibulated women obtained significantly higher scores in desire, arousal, orgasm, and satisfaction.
Alsibiani and Rouzi^40^	Case–control study	Saudi Arabia	260 (*n*=130 infibulated women; *n*=130 unmutilated group; both groups enrolled exclusively sexually active women)	21.4±4.4 in the FGM/C group vs. 23.5±5 in the control group	Significantly lower full-scale score in the FGM/C group. No statistically significant group differences in desire and pain scores. Statistically significant lower arousal, lubrication, orgasm, satisfaction, and lubrication in the FGM/C group
Anis et al.^41^	Cross-sectional comparative study	Egypt	650 (*n*=333 women with FGM/C; *n*=317 women without FGM/C)	23.9±2.2 in the FGM/C group vs. 26.8±2.2 in the control group	Significantly lower full-scale score in the FGM/C group. All domain scores, except for sexual pain, were significantly lower in the FGM/C group as compared with those of the uncut women.
Ibrahim et al.^42^	Cross-sectional study	Egypt	509 (*n*=365 women with FGM/C)	20.1±3.5 (including women with and without FGM/C)	65% of the FGM/C participants reported sexual dysfunction. Circumcision was the leading factor associated with sexual dysfunction (OR 6.5, 95% CI: 2.6–15.8)
Mohammed et al.^43^	Cross-sectional study	Egypt	2106 (*n*=1911 women with FGM/C; *n*=195 women without FGM/C)	29.6±2.1 in type I FGM/C, 10.7±3.4 in type II FGM/C, and 34.2±0.3 in the non-FGM/C group	Desire, arousal, lubrication, orgasm, and satisfaction were significantly poorer in women with type II FGM/C. Pain was significantly higher in type II FGM/C.
Abdulcadir et al.^44^	Cross-sectional study	Switzerland	30 (*n*=15 women with FGM/C; *n*=15 women without FGM/C)	27.0±3.1 in the FGM/C group vs. 30.7±4.2 in the control group	Significantly lower full-scale score in the FGM/C group. No significant differences between the two groups in desire, orgasm, and satisfaction. Women with FGM/C reported significantly lower arousal and lubrication, and greater pain relative to uncut women.
Biglu et al.^45^	Case–control study	Iran	280 (*n*=140 women with FGM/C; *n*=140 women without FGM/C)	17.9±5.3 in the FGM/C group vs. 25.3±4.3 in the control group	Significantly lower full-scale score in the FGM/C group. All domains were significantly lower in the FGM/C group as compared with uncut women.
Mahmoud^46^	Case–control study	Egypt	544 (*n*=272 women with FGM/C; *n*=272 women without FGM/C)	14.3±5.9 in the FGM/C group vs. 25.9±3.4 in the control group	Significantly lower full-scale score in the FGM/C group. All domains were significantly lower in the FGM/C group relative to those of the uncut women.
Vital et al.^47^	Prospective	France	12	17 (IQR: 13–21) (before surgical reconstruction) vs. 29 (IQR: 24–34) (6 months after surgery)	Desire, arousal, orgasm, and pain were the most affected domains before surgical correction. Significant improvement of FSFI full-scale score after surgery. The ameliorations were significant in all subdomains, except for lubrication.
Rouzi et al.^48^	Cross-sectional study	Saudi Arabia	107	21.2±6.37 (26.8±1.9 in type I; 21.6±2.8 in type II; 14.9±5.5 in type III)	Nine out of 10 women with FGM/C suffered from sexual dysfunction. Women with type III FGM/C showed the worst scale scores.
Ismail et al.^49^	Case–control study	Egypt	394 (*n*=197 women with FGM/C type I and II; *n*=197 women without FGM/C)	19.8±7.1 in the FGM/C group vs. 23.3±8.1 in the control group	Significantly lower full-scale score in the FGM/C group. All domains were significantly lower in the FGM/C group relative to those of the uncut women. No statistically significant difference between the two types of FGM/C as regards total and individual domain scores except for the pain domain.
Daneshkhah et al.^50^	Cross-sectional study	Iran	200 (*n*=140 women with FGM/C; *n*=60 women without FGM/C)	18.2±6.3 in the FGM/C group vs. 23.9±7.1 in the control group	Significantly lower full-scale score in the FGM/C group. All domains were significantly lower in the FGM/C group relative to those of the uncut women.
Esho et al.^51^	Cross-sectional study	Kenya	314 married women (*n*=140 women with FGM/C cut before marriage; *n*=29 women with FGM/C cut after marriage; *n*=145 married women without FGM/C)	23.9±6.6 in the FGM/C group (cut before marriage) vs. 22.8±4.9 in the FGM/C group (cut after marriage) vs. 25.3±3.5 in the control group (married and uncut)	Women cut after marriage scored significantly lower than the uncut. No statistically significant difference between the two FGM/C groups. Among the sexual functioning domains, lubrication, orgasm, and satisfaction were significantly different across the three groups. Desire, arousal, and pain were not statistically different.
Manero and Labanca^52^	Prospective	Spain	32 (*n*=4 women with FGM/C type I; *n*=25 women with type II; *n*=3 women with type III)	16 (IQR: 12–21) (before surgical reconstruction) vs. 29 (IQR: 26.1–31.2) (6 months after surgery)	Significant improvement of FSFI full-scale score after surgery. The ameliorations were significant in all subdomains, except for desire.

CI, confidence interval; FGM/C, female genital mutilation/cutting; FSFI, Female Sexual Functioning Index; IQR, interquartile ranges; N.A., not applicable; OR, odds ratio.

Overall, although not all women with FGM/C show sexual issues, it seems that they may be physiologically less capable of becoming sexually stimulated than uncut women,^26^ due to the essential role of the integrity of the clitoris and labia minora for the achievement of sexual response.^36^ It is important to specify that in women with FGM/C some essential structures involved in the achievement of orgasm have not been removed. In fact, anatomically, the mutilation consists in the excision of the externally visible portion of the clitoris (the glans), whereas the crura and the body remain intact under the scar.^44^ Nour et al.^54^ found an intact clitoris in 48% of 40 women undergoing defibulation.

In addition, another possible compensatory mechanism to overcome the “anatomical barrier” is the ability of women to enhance stimulus originating from other sensory or erotic areas, or through the ideation of emotions and fantasy.^26^ Mutilated women identify their breasts, tongue, or vagina as their most sensitive parts of the body.^55–57^ Moreover, as suggested by Thabet and Thabet^36^ sexual function in women with FGM/C is the result of multiple cofactors, including a correct sexual and genital knowledge, and an adequate sexual stimulation to achieve a satisfactory response.

Another important question is the existence of a dose–response relationship between FGM/C and sexual functioning. One can hypothesize that the more severe the woman's excision is, the greater the consequences are.^2^ This theory was supported by Andersson et al.,^53^ who stated that, in sexually active women, type III FGM/C was associated with the lowest sexual quality of life scores as compared with controls. Mohammed et al.^43^ found significantly worse sexual functioning in women with type II FGM/C relative to women with type I FGM/C and those without FGM/C. A recent cross-sectional study^48^ also demonstrated that women with type III FGM/C presented the worst FSFI scale score. Based on these findings, a firm conclusion on this topic is not yet achievable. Future research should compare women with different types of FGM/C to elucidate the possible dose–response relationship involved in FGM/C and sexual functioning.

## Reconstructive Surgery

Clitoral reconstruction is a surgical technique first described by Thabet and Thabet^36^ in the early 2000s. The procedure consists in the resection of the skin that covers the stump with the aim of revealing the clitoris; the suspensory ligament is subsequently sectioned to mobilize the stump. The scar tissue is then removed, and the glans is placed into a physiological position.^58^ Surgery is usually performed under general anesthesia to avoid the patient from reliving the traumatic experience of FGM/C.^58^ The aim of surgery is to restore both clitoral anatomy and function, to improve patient's self-esteem, body image, sexual function, and reduce pain during sexual intercourse.^59^

Prospective studies of the impact of reconstructive surgery on the sexual function of women with FGM/C are limited.^36,47,58,60^ Foldès et al.^58^ published a large prospective study on 2938 women with FGM/C who underwent clitoral reconstruction. In the vast majority of the patients (>99%), the primary expectation for surgery was the recovery of identity, followed by an improvement of sexual life (81%) and pain reduction (29%). Immediate complications after surgery, including hematoma, suture failure, and moderate fever, were registered in 5.3% of the patients, and 3.7% were readmitted to hospital. The 1-year follow-up was completed in 841 patients (29%). After surgery, one woman out of three (35%, *n*=129/368) who had never experienced orgasm before surgery began to have restricted or regular orgasm, and half of the women who had restricted orgasm before surgery reported a regular orgasm after the procedure. In addition, 97.7% of the participants reported a decrease in pain, and a visible glans was observable in 70% of the patients. In particular, the latter category of patients was 2.2 times more likely to experience orgasms than those without a visible glans (95% CI: 1.40–3.43). However, 23% (*n*=12/53) of the women who were able to regularly reach orgasm before surgery reported a reduction in orgasmic frequency after surgery. These results are in line with those of Vital et al.,^47^ who performed clitoral reconstructive surgery on 12 women. In this prospective study, a validated questionnaire, the FSFI, was used to assess the impact of surgery on sexual function. At 6-month follow-up, women showed a multidimensional positive improvement in their sexual function, with a significantly higher FSFI full score (Table 3). In addition, 11 out of 12 women were satisfied with the procedure and the appearance of their genitalia and sense of femininity.

A recent systematic review^61^ evaluated the effects of reconstructive surgery. The results indicate that about three women out of four regain a visible clitoris; self-reported ameliorations in pain during sex, clitoral function/pleasure, orgasm, and desire are in the 43–63% range, but up to 22% reported a worsening in sexual outcomes. As underlined by the authors, it is difficult to ascertain the real impact of reconstructive surgery due to methodological limitations and insufficient study similarity.

Clitoral reconstruction represents the principal but not the only reconstructive option for women with FGM/C; other possibilities include reconstruction of the clitoris and labia, defibulation, removal of cysts, neuromas, and scar tissue.^62^ In addition, these approaches can be combined with novel reconstructive techniques. Chang et al.^63^ performed on three consecutive women with grade II FGM/C a new clitoral reconstruction technique based on the concept of anchoring the labia majora to the pubic bone to reduce the risk of labial fusion. In 2018, a Spanish study^52^ described a novel surgical technique for clitorolabial reconstruction using a vaginal graft. A total of 32 consecutive women were enrolled, no intraoperative or postoperative complications were encountered, and at 6 months follow-up a statistically significant improvement of FSFI score, as well as a favorable change in the Female Self-Image Genital Scale, was registered.

## Conclusion

Although it is clear that FGM/C can cause devastating immediate and long-term health consequences for girls and women, high-quality data on these issues are limited. In particular, psychosexual complications need to be further analyzed to provide evidence-based guidelines and to improve the health care of women and girls with FGM/C. In addition, the perception and experiences of FGM after immigration in Western countries should be further investigated, to explore the point of view of the women themselves.

Sexual health represents a fundamental aspect of individual well-being, and sexual dysfunction is related to significant personal distress.^64^ Thus, gynecologists and other clinicians should assess the sexual functioning of women with FGM/C, and propose a specific personalized approach when dysfunctions are ruled out. Furthermore, besides pain due to anatomical distortion related to the genital mutilation, psychological dimensions (such as anxiety, depression, PTSD, as well as sense of female identity) or relational mechanisms (feelings of shame, behaviors, marital dissatisfaction) play a central role in sexual functioning.^65,66^ Consequently, a comprehensive approach considering all the various aspects of female sexuality should be encouraged. As suggested by WHO guidelines,^1^ sexual counseling should be proposed for preventing or treating sexual dysfunctions among women with FGM/C.

Several studies have demonstrated that there is a connection between sexual pleasure and the ideas a woman has concerning her genitalia.^67,68^ From this perspective, if a woman is worried about the aspect of her external genitalia, this can lead to negative consequences, such as shame and reduction of self-esteem, with repercussion on her sexual sphere.^69,70^ In these cases, careful counseling and a multidisciplinary approach are essential to identify those women who would most benefit from a surgical and/or psychological approach.

Clitoral reconstruction may also represent a valuable option to reduce chronic clitoral pain by excising scar tissues and improve sexual function among women who have undergone FGM/C.^58^ In the postoperative recovery time, an important period of transition, follow-up is central, as well as psychosexual support. However, some concerns have been raised about the potential negative consequences—either physical or psychological—of this type of surgery.^71^ To date, there are no guidelines recommending clitoral reconstruction. The Green-top Guideline from the Royal College of Obstetricians and Gynaecologists^72^ on the management of women with FGM/C concluded that further studies are necessary before recommending this procedure, and future trials should evaluate the safety and efficacy outcomes in the long-term period using validated and standardized tools.^71^

It is important to inform women seeking reconstructive surgery of the paucity of evidence on the safety and long-term efficacy of the procedure.^71^ In conclusion, FGM/C requires personalized treatment, in which the best options should be identified and indicated according to women's individual problems, preferences, and priorities.

## Abbreviations Used

CBT: cognitive behavioral therapy
CS: caesarean section
FGM/C: female genital mutilation/cutting
FSFI: Female Sexual Functioning Index
PTSD: post-traumatic stress disorder
SQOL-F: Sexual Quality of Life-Female
WHO: World Health Organization
